# Enhancing System Acceptance through User-Centred Design: Integrating Patient Generated Wellness Data †

**DOI:** 10.3390/s22010045

**Published:** 2021-12-22

**Authors:** Sarita Pais, Krassie Petrova, Dave Parry

**Affiliations:** 1 Department of Computer Science & Software Engineering, Auckland University of Technology, Auckland 1010, New Zealand; krassie.petrova@aut.ac.nz; 2 Department of IT, Media and Communications, Murdoch University, Perth 6150, Australia; david.parry@murdoch.edu.au

**Keywords:** gestational diabetes mellitus, user-centred design, system acceptance, TAM, UCD, think-aloud protocol, mHealth, mobile app

## Abstract

Gestational diabetes mellitus (GDM) is a condition that appears during pregnancy and is expected to be a temporary one. While patients are encouraged to manage it themselves, research findings indicate that GDM may negatively affect the foetus; in addition, there is an increased risk of women with GDM subsequently developing Type 2 diabetes. To alleviate the risks, women with GDM are advised to maintain a record of their diet and blood glucose levels and to attend regular clinical reviews. Rather than using a paper diary, women with GDM can maintain a record of their blood glucose level readings and other relevant data using a wellness mobile application (app). However, such apps are developed for general use and may not meet the specific needs of clinical staff (physicians, dietitians, obstetricians and midwives) involved in managing GDM; for example, an app may record glucose readings but not the details of a meal taken before or after the glucose reading. Second, the apps do not permanently store the data generated by the patient and do not support the transfer of these data to a clinical system or information portal. The mobile health (mHealth) system designed and developed in this research allows one to integrate different types of user generated wellness data into a centralised database. A user-centered design (UCD) approach informed by the technology acceptance model (TAM) was adopted. This paper investigates and evaluates the effectiveness of the approach with regard to facilitating system acceptance and future adoption through an early focus on enhancing system usefulness and ease of use. The functional system requirements of the proposed system were refined through a series of interviews with the perspective of clinical users; ease-of-use and usability issues were resolved through ‘think aloud’ sessions with clinicians and GDM patients.

## 1. Introduction

Worldwide, the prevalence of gestational diabetes mellitus (GDM) varies and can be up to 28% in certain populations [1]. In the last 10 years, the number of women affected by GDM has grown, for example, in multi-ethnic cities; in some ethnic groups (Indian, Asian and Pacific) the prevalence of GDP is 22% higher, compared to Europeans [2]. The condition appears during pregnancy and is usually temporary. While patients are encouraged to manage it themselves, research findings indicate that GDM may affect negatively the foetus; in addition, there is an increased risk of women with GDM subsequently developing Type 2 diabetes [3].

The benefits of monitoring blood glucose and adhering to a prescribed dietary plan as factors decreasing maternity and pregnancy risks have been shown in medical trials involving women with GDM [4]. Therefore, GDM self-management requires changes in the patient’s lifestyle, including diet and physical activity [5]. However, to alleviate the risks to their pregnancy, women with GDM are advised to maintain a record of their diet and blood glucose levels and to attend regular clinical reviews. 

Traditionally, women were asked to keep a diary and share the information recorded at consultation time. Today, mobile health (mHealth) solutions have shown promising results for better self-management of blood glucose and diet [6,7,8,9]. For example, women with GDM can keep records showing blood glucose readings and exercise routine details, and maintain a food diary with the help of the many health and wellness mobile applications (wellness apps), available on both Android and iOS mobile devices. Moreover, quite a few of these apps are capable of sharing patient diaries and recordings with patient-authorised third parties, for example, health professionals, by email or through a web interface [6]. 

Clinics have already started trialing the use of such apps at consultation time. However, the use of the data provided by the apps is very limited as the apps do not store them permanently and do not support the transfer of these patient data to a clinical system or information portal. Both GDM patients and consulting clinicians would benefit from having access to a health system that stores vital GDM patient data such as periodic glucose readings and can make them available for further reference and review. Such a system would contribute effectively to maintaining high standards of health care and continuity. The mHealth prototype described by Pais et al. in [10] addressed the issue above by creating a clinical ecosystem that integrated glucose level, food intake, exercise and medication (insulin) data generated by commercially available wellness apps (further referred to as ‘patient-generated wellness data’ or ‘wellness data’) into a centralised database. The ecosystem aims to assist midwives as well as women with GDM in managing the condition and to provide the input needed by specialist health professionals (i.e., obstetricians and dieticians) when recommending diet and medication. 

The ecosystem design and development were informed by research in the area of information technology adoption and, in particular, by the technology acceptance model (TAM). A comprehensive review of theories and models explaining the adoption and acceptance of new technology can be found in [11]. According to the findings, TAM, along with UTAUT (unified theory of acceptance and use of technology) and DOI (diffusion of innovations theory), are the three most frequently used models. Harst et al. [12] also found that TAM and UTAUT were the most widely used models used to investigate telemedicine adoption. A common assumption underlying these models is that environmental constructs such as information technology artefacts influence individuals’ cognitive beliefs (i.e., beliefs that are based on the information the individual holds about the particular environmental construct). Therefore, TAM and UTAUT can provide an appropriate theoretical framework for the analysis of data gathered from information system end-users as they observe and interact with the information technology artefact, as part of the user-centred design (UCD) process [11,12].

According to TAM, the acceptance and actual use of a socio-technical system is mediated by system usage intention, influenced by individual users’ perceptions of the system’s usefulness (functional capabilities) and ease of use (usability) [13,14]. TAM has been validated in empirical mHealth research as a means of subjective system evaluation that can provide deep and meaningful insights on how users interact with technology [15,16]. Tao et al. [17] also concluded that TAM was a ‘good ground theory’ for the investigation of the user adoption of consumer-oriented health information technologies. Acknowledging that user acceptance of patient-centred technology supported systems may still present a challenge [14], gather and analyzing user feedback about the clinical ecosystem functionalities and usability was a particular focus of this research. 

In health systems, UCD has been applied as an approach that facilitated system acceptance and promoted actual system use by the target users. For example, Sobrinho et al. [18] developed a prototype of a system (a mobile app) that aimed to assist patients in the self-management of chronic kidney disease; users (i.e., patients and health professionals) were involved through a UCD approach that deployed in-depth interviews as a data collection method. Similarly, Calvillo-Arbizu et al. [19] adopted a UCD approach to build a health system that enabled monitoring home-based renal patients. Patient, caregiver and clinical professional views and opinions about the system functionalities were captured through interviews and questionnaires; the stakeholders were involved in the periodic revisions of the incrementally developed prototype. More recently, De Luca et al. [20] applied a UCD approach towards the development of a regional digital service for integrated hypertension management. Interview data gathered from patients and health professionals were used to define the functional requirements of the system and to identify its use cases.

The UCD process is an iterative one, with users and other stakeholders providing feedback and evaluating system design decisions in a loop that starts with the initial prototype and continues till the final version of the system is produced. Developing health systems applying a UCD approach aims to improve system functionality and usability, and consequently, to achieve better clinical outcomes [21]. To ensure user acceptance, it is important to engage with the diverse body of clinical systems users early in the design process and to periodically and systematically gather stakeholder feedback about system usefulness and usability [22,23]. Thus, the overarching research question of the study presented here is formulated as follows: “How to ensure that the system design process of a clinical ecosystem that integrates patient generated wellness data facilitates user acceptance?”. 

In this study, the system design and development process made certain that users of the ecosystem shared a common understanding of how the system would be used and what their respective tasks would be, and what data were needed to facilitate and support GDM patient self-management. The UCD approach ensured that the system’s functionality met the requirements of the principal system users. Second, the iterative design process provided multiple opportunities to address usability issues raised by participating users [22].

The flexibility of the UCD process allowed one to involve users by deploying a range of techniques and methods [21]. Clinicians caring for women with GDM in a variety of roles were identified as principal system stakeholders. Dieticians, obstetricians and midwives had the opportunity to be part of the design of the prototype right from the initial stages; they took part in identifying the system requirements and reviewed the prototype iterations. Women with GDM were also invited in the later iterations as they are responsible for the self-management of their condition and were required to provide input to the health system by sharing their blood glucose and diet details.

The rest of the paper is organised as follows. The next section introduces a conceptual view of the clinical ecosystem and describes the study participants and the gathering of data and analysis methods used. The findings and their implications are presented and discussed in the following two sections. The last section summarises the results, outlines the study limitations and provides directions for further research. 

## 2. Materials and Methods

### 2.1. Clinical Ecosystem Architecture 

**Figure 1:**
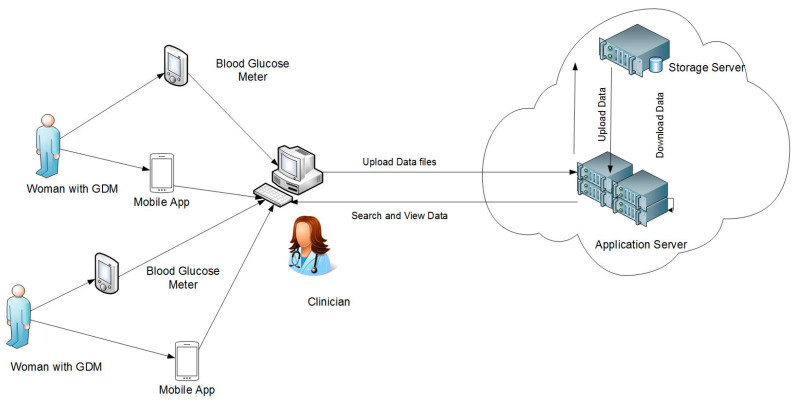
Clinical ecosystem integrating patient generated wellness data: a conceptual view.

A conceptual model of the architecture of the prototype ecosystem and the stakeholder interactions is shown in Figure 1. The ecosystem’s database integrated four types of data: blood glucose readings, data about food intake, data about physical exercise and data about medication (insulin). The four types were determined based on prior studies about managing women with GDM [3,5,8] and on the paper-based forms used by the staff at the host diabetes clinic. Therefore, the functional capabilities of the system included extracting, downloading and storing data about blood glucose readings, food intake (food diary), physical exercise, and inulin dosage, from the remote devices used by women with GDM (mobile devices and glucose meters).

The mobile apps that were included in the prototype were selected based on their capability to keep a record of blood glucose readings, exercise and diet, and to have provisions for exporting the data. Maintaining a medication log was desirable but not compulsory as medication data were also available at the clinic. With regard to the food diary, the app needed to be able to record not just the calories but also details such as the name of the food item, the portion size and the main nutrient content. Only commercially available apps were considered.

**Figure 2:**
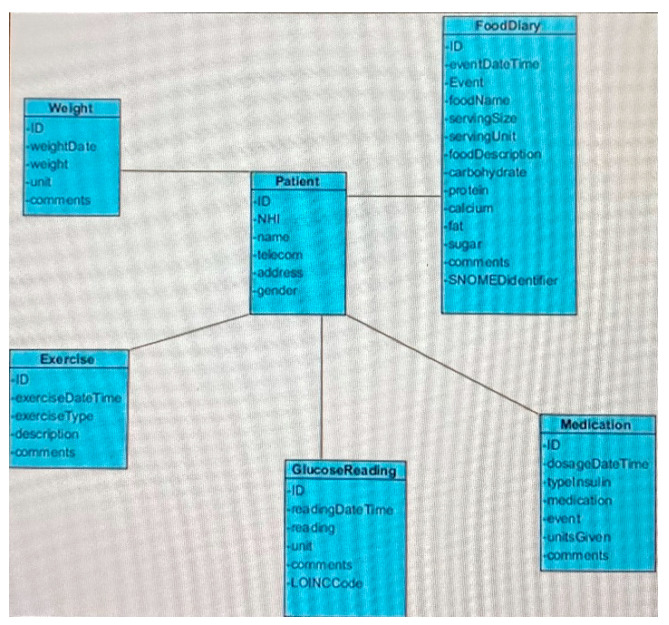
Database schema.

The data elements for storing the downloaded patient data are shown in the database schema in Figure 2. It is sufficiently flexible to allow expanding the ecoystsem’s scope by adding new apps and devices. 

An initial search for health and wellness apps showed their number as extremely high and that the descriptions that could be found were not always complete and clear, if present at all. As the research did not aim to study comprehensively health and wellness apps, it was decided to apply a purposive approach and identify apps based on reputable recommendations and descriptions. The apps need to match the search criteria; other properties and data output were ignored.

A starting point for the search for suitable commercially available apps was the website of the non-profit community service Health Navigator, which lists and compares apps recommended for the self-management of diabetes type 2 (https://www.healthnavigator.org.nz/apps/d/diabetes-type-2-apps/, accessed on 10 December 2021). The apps reviewed in [6,8] were also considered; however, they did not meet the selection criteria (food diet data were not recorded). Further search included other recommended websites and well-known app stores. 

**Figure 3:**
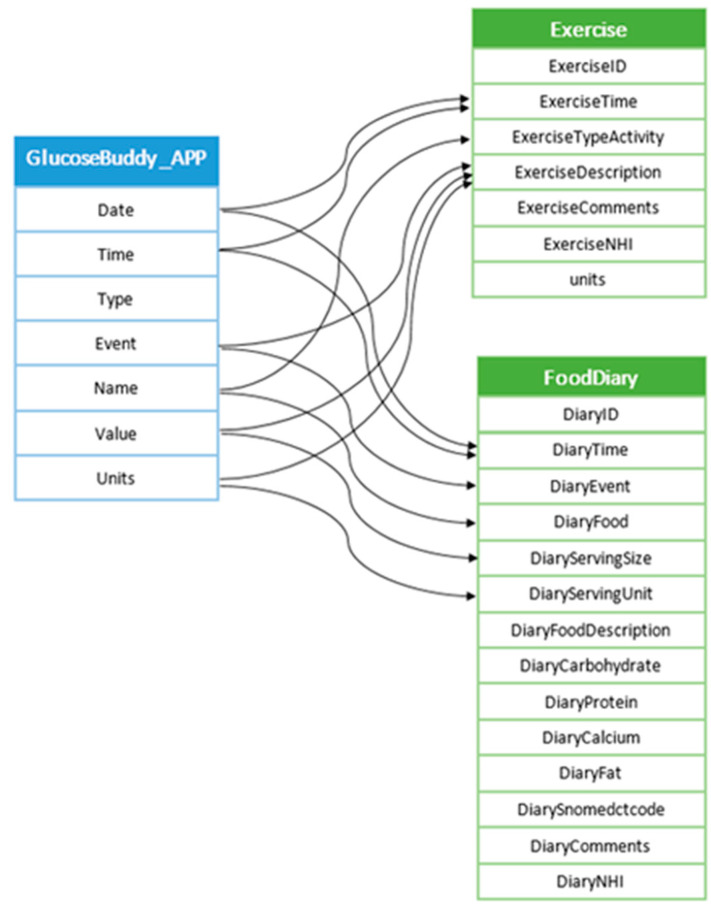
Mapping exercise and food diary data generated by the Glucose Buddy app.

Two apps were initially selected and integrated in the prototype: On Track and Glucose Buddy (available for both Android device and iPhone); Glucose Buddy was one of the apps recommended by Health Navigator. Both apps maintained logs of blood glucose readings, and food intake and physical exercise. Glucose Buddy also recorded medication details. Both apps used the CSV (comma separated values) format for sharing data. CSV is simple non-proprietary that supports app interoperability; it provided a straightforward way of transferring the data to the integrated database. However, each app had its own data schema, and it was necessary to write app-specific code that mapped the data fields of the extracted app data to the data schema of the clinical system (manual mapping). The example in Figure 3 shows the mapping of the food and exercise data generated by Glucose Buddy to the integrated database. 

Three more apps were added later: mySugr and Easy Diet Diary (both recommended by Health Navigator), and My Meal Mate (developed by a team from the University of Leeds, UK). The apps used non-proprietary data standards (XML, CSV, SQL). All apps generated glucose readings, food intake and exercise data. mySugr had an option for maintaining a medical diary. In addition, the app was already familiar to the participating clinical specialists as some of their Type 1 diabetes patients used it to show personal wellness data on the screens of their smart phones. The two other apps (My Meal Mate and Easy Diet Diary) were particularly suitable as each had a built-in food database and it was easy for the patient to maintain their food diary. 

The popular app MyFitnessPal was also considered as it generated the patient data needed for the clinical ecosystem and had a food database. However, only ‘premium’ account holders had the option to share data file. Even though the app could be used with devices such as Fitbit, the API was proprietary. While MyFitnessPal was successfully included as a trial, it was dropped from the prototype as potentially costly for patients.

**Figure 4:**
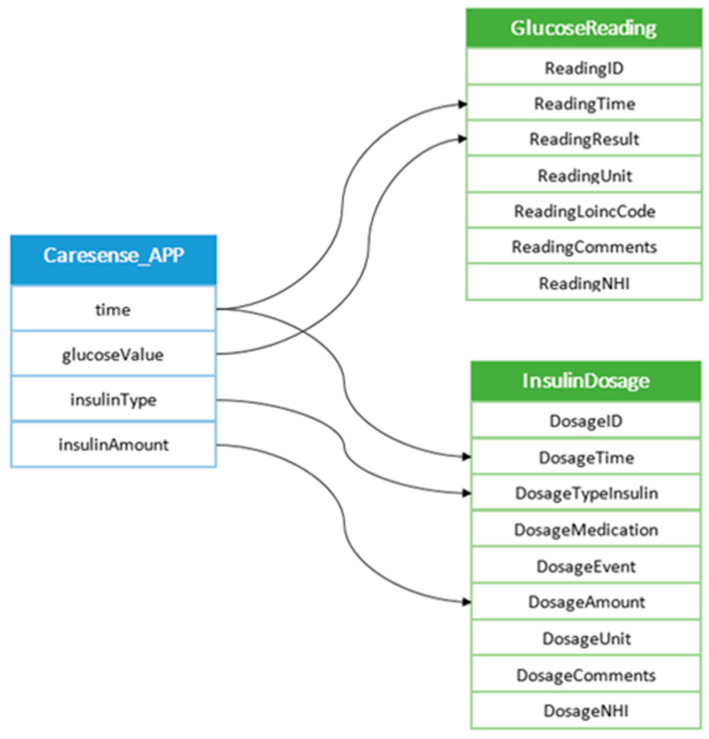
Mapping glucose readings and insulin data from the glucose meter (Caresense).

The clinical system database also accommodates the data exported by the glucose meter Caresense, which were provided to the patients by the clinic. (The glucose meter is not integrated into the hospital or clinic systems. A standalone PC is used in the clinic to download patient readings.) The example on Figure 4 shows the mapping of the data generated by the meter onto the database schema. 

**Figure 5:**
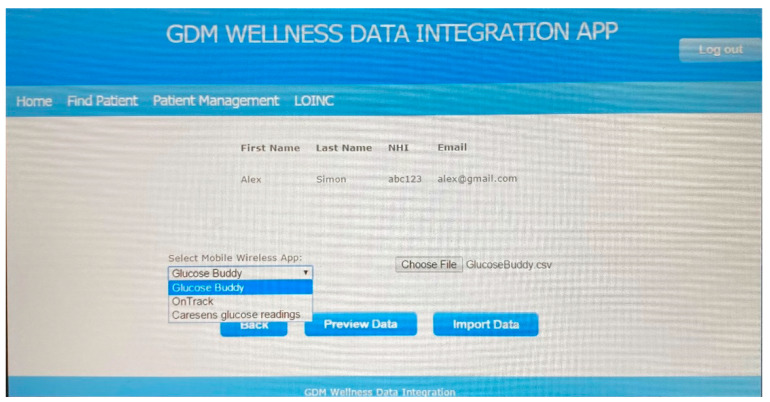
Downloading patient data.

The screenshot in Figure 5 shows how a clinician can download the data of the fictitious patient Alex Simon, generated by the app Glucose Buddy. The clinician has to login first, then find and select the patient (Alex Simon) and identify and select the mobile app used by the patient (Glucose Buddy) and the app output file shared with the clinic (GlucoseBuddy.csv). Once the ‘Import’ button is clicked, the selected wellness data are downloaded and integrated with the rest of the patient’s data stored in the prototype database. 

As seen in Figure 5, the prototype enabled clinicians to access patient data stored in the integrated database after they were authenticated as system users (by their login name and password). It was assumed that the security layer of the prototype would be developed further at implementation time. It was also assumed that protecting data stored on mobile phones would be the responsibility of the patients who opted to use a mobile wellness application.

### 2.2. Study Participants 

Particular care was taken to ensure that this study took into account the diversity of the roles of collaborating clinical user groups, as also recommended by Shachak et al. [24]. At a diabetes clinic, women with GDM normally have regular appointments with their midwives, who also review the data about blood glucose, food intake and physical activity that are provided by the patient. The data may be reviewed as well at appointments with specialist clinical staff (obstetricians, dieticians and physicians). Therefore, midwives, obstetricians, dieticians and physicians were identified as the target clinical users of the ecosystem, while women with GDM were the target patient users. Therefore, research participants were recruited from amongst the relevant clinical staff and patients of a diabetes clinic that agreed to host the research. 

Invitations to participate in the research were displayed on notice boards accessible to clinical staff. Interested clinicians were invited to write to an email address provided in the invitation, within a week. In reply, they were sent back an information sheet and a consent form. Two recruitment rounds were carried out; participants were randomly selected from those who responded to the invitations.

Staff at the clinic were given printed invitations, which they agreed to distribute to women with GDM. Patients interested in participating in the research were asked to reply to the email address provided and were subsequently sent an information sheet and a consent form. Participants were selected randomly from the ones who responded to the invitation.

As described in Table 1, the study data were collected through semi-structured interviews and live think-aloud (TA) sessions. The latter may require significant labour and time commitment; due to pragmatic considerations, research participant samples may be kept relatively small and purposefully selected [25]. In this study, the research sample represented all principal stakeholders. It comprised 15 individuals: ten clinicians and five patient participants were recruited. Similar sample sizes have been used in empirical research involving TA as a data collection method. For example, 13 health professionals participated in Kilsdonk et al.’s study [26] and 12 health care providers in Richardson et al.’s study [27].

The semi structured interviews were conducted alongside the TA sessions and involved the same participants. As the study’s aim was specific rather than broad and data were gathered from participants associated with one clinic only, the ‘information power’ of the sample was deemed adequate [28].

The clinical research participants actively involved in the first three evaluation stages (Table 2) included two dieticians, two obstetricians and one midwife. A physician also participated by providing additional operational information to guide the design. These participants provided requirements and reviewed the initial ecosystem version (Prototype 1). Five other clinicians (midwives) and five women with GDM were further recruited to evaluate the second version of the prototype (Prototype 2), where the requirements about the scheduling of blood glucose readings, and determining the relevant insulin dosage, were reviewed by all obstetricians and midwives. The food and exercise details were also reviewed by all dieticians and midwives. 

### 2.3. Data Gathering and Analysis Methods 

The ‘live’ data gathered at a TA protocol session reflect the immediate reactions of the research participants as they perform and comment on the tasks prescribed by the researcher. These tasks are modeled on some of the systems use case scenarios; the objective is to give users an opportunity to engage with system and by doing this, identify latent usability issues. Normally, the audio recordings of TA sessions are complemented by video recordings of the systems screen. As participants are encouraged to freely verbalise their experience and provide a commentary, system developers may be able to discern potential solutions to the problems raised. 

Overall, the analysis of the TA data may provide the researcher with useful insights about how users perceive the system they are interacting with [29]. For example, data gathered through TA protocol sessions have been used to provided substantial input with regard to the design and development of the clinical decision support systems reported in [26,27], and to pinpoint patients’ concerns with the usefulness and usability of a health information system [30]. 

Using a multimodal data gathering approach (e.g., combining TA with another data collection method) as a means of obtaining rich user feedback may help consolidate the differing and at times contradicting views and opinions of the target health information systems users [31]. For example, Money et al. [32] conducted interviews and applied the TA protocol to evaluate a computer application for designing a home environment suitable for the installation of safety equipment for older patients transferring from hospital to home care. Similarly, Cervera et al. [33] used TAM and the TA protocol to measure the perceived usefulness (PU) and perceived ease of use (PEOU) of a new system and obtained consistent results for PU and PEOU using the two methods. 

The study combined the use of the TA protocol with semi-structured interviews to gather further qualitative data, and applied content analysis to identify the emerging main and sub-topics relevant to the research question [34]. Pre-distributed video and documentary material were also used to help participants understand better the design of the prototype. The study applied TAM as a framework for the analysis of subjective user feedback, by developing inductively themes around the ecosystem’s usefulness and usability/ease of use. 

### 2.4. System Evaluation Process 

Stakeholder feedback on system usefulness and usability/ease of use was gathered and explored throughout the design process. Following prototype demonstrations, participants were given an opportunity to check the functional capabilities of the system, including the correctness of the mapping of the data captured from the wellness apps onto the prototype global schema, and to validate the content and the design of the generated clinical reports. To facilitate a better understanding of the clinical ecosystem’s concepts and functionalities, participants were sent a user manual (for each iteration of the prototype, and prior to the interviews and the TA sessions). The evaluation included five major evaluation stages: two ex-ante stages (i.e., before developing Prototype 1) and three ex-post stages (i.e., after developing Prototype 1). At each stage, participants were given demonstrations and were provided with opportunities to express their views and opinions, and to suggest changes. As shown in Table 2, at the ex-ante evaluation stages data were gathered through two rounds of semi-structured interviews (INT1 and INT2), while the ex-post evaluation rounds involved one semi-structured interview round (INT3) and two TA sessions (TAP1 and TAP2).

During the semi-structured interviews, clinicians provided feedback about Prototype 1 and Prototype 2 by drawing on their experience of supporting their patients in the self-management of GDM. The recorded interview data were transcribed and analysed qualitatively. The average interview length was about 40 min. 

At the TA sessions, participants engaged with the protype by completing a series of prescribed tasks, at the same time verbalizing their actions. TAP1 tasks included: (i) view combined wellness report (subtasks Login as a clinician, Find patient and View combined report for a range of dates); and (ii) import wellness data (subtasks Find patient, Import data and View combined report for a range of dates). TAP2 tasks included the same tasks as TAP1 plus three new tasks; (iii) identify fasting and non-fasting readings and assign LOINC codes (subtasks Find patient, View blood glucose readings for a range of dates and Identify whether fasting or non-fasting); (iv) assign SNOMED CT code for food item (subtasks Find patient, View food diary for a range of dates and Assign SNOMED CT code); and (v) assign nutrients value (subtasks Find patient, View food diary for a range of dates and Select food item to get calculated nutrients value). Participants’ utterings were recorded along with a video capture of the screen they were working on. The transcribed audio records were analysed qualitatively. A typical TA session lasted about 45 min. The feedback gathering activities at each stage are described below.

1. Open ended semi-structured interviews (INT1) with five participants were conducted to elicit the requirements of the system prototype. Paper mockups of the interface design and navigation were prepared and discussed with the participants. A requirement analysis document was created and shared with the participating clinicians by email. It included screenshots of the interface design, a review of the selected mobile apps and a conceptual description of the database design, including the wellness data to be stored.
2. Videos of a prototype demonstration were created and sent to clinicians. A user manual presenting the main functionalities of the prototype illustrated with screenshots was also distributed. The second set of interviews (INT2) with clinicians was organised. The prototype was demonstrated interactively to individual participants, who could query the functionalities and the design of the prototype. Usability issues were highlighted at this stage.
3. A TA session (TAP1) was conducted to check whether participants (clinicians) were comfortable undertakings basic tasks such as uploading wellness data into the system and viewing the system outputs as a series of combined daily reports containing blood glucose levels, a food and exercise diary, and insulin dosage. Further usability issues were raised.
4. A third set of interviews (INT3) was organised, involving the clinicians who had participated so far, five other clinicians and women with GDM. The prototype was demonstrated to those individuals who were new to the system. The demonstration was interactive, with participants exploring the functionalities and the design of the prototype.
5. A second TA session (TAP2) was conducted. Its first aim was to check whether the new group of participants (the clinicians and women with GDP who participated in INT3) were comfortable undertaking the basic tasks already introduced at the TAP1 session. Second, participating clinicians were asked to complete two new tasks which aimed at enhancing the clinical value of the data (e.g., adding a nutrition value to a food item).

It was somewhat difficult to arrange meetings with clinicians who were busy with their regular clinical consultations. Most meetings were arranged between patient consultation appointments at the clinic. While it was not appropriate to be present during the consultations due to privacy issues, the authors were given the opportunity to observe the practice at the clinic and meet women with GDM.

## 3. Results

The qualitative data gathered at each of the evaluation stages searched for meanings related to the TAM variables PU, PEOU and BI (behavioural intention to use). The resulting data clusters were re-examined and coded inductively to identify the emerging themes around system usefulness and system ease of use. 

### 3.1. System Usefulness and User Acceptance 

The aim of the first evaluation stage was to formulate the key functional requirement of the system. The results of the analysis of the data gathered at this stage (i.e., INT1 data and feedback on the requirements analysis document) indicated that, according to clinicians, using smart phone technology had the potential to enhance the GDM self-management process. Even though clinicians were somewhat reserved about adopting new technologies, they were quite interested in a system that integrated GDM patient data generated by wellness apps installed on patient mobile phones. Participants identified features that they thought would be useful: all relevant patient data presented in a single report, with blood glucose readings and food intake items described in sufficient detail.

- All data in one report: in current practice, data about blood glucose levels, food intake (food diary), exercise and insulin dosage are generated by different sources and communicated through different channels (e.g., on paper, or in an email). The new system would be useful if it collated these data and presented them in one single report.
- Blood glucose reading labeled: blood glucose reading data were not always precise about timing (e.g., after or before a meal). The new system needed to provide these details and highlight readings above the norm.
- Food portion size: the system needed to capture data about the size of the food portion (not only about the food type) and to provide for adding the relevant LOINC code to improve accuracy.
- Food description: the apps that generated the wellness data needed to contain food descriptions, to enable accurate and effortless data entry.
- Exercise data needed to be captured, if provided by a wellness app.
- Weight data needed to be captured, if provided by an app (or entered manually).
- Provide for adding SNOMED terms to food items to enable exporting patient-generated wellness data to other clinical systems.

User feedback about the usefulness of the ecosystem prototype was the data gathered mostly through INT2 and INT3. In INT2, the five participating clinicians were asked to consider using the system in their everyday practice, based on a visualisation of Prototype 1. In INT3, the system’s usefulness was evaluated by ten clinicians and five women with GDM who observed Prototype 2. Twelve system usefulness themes emerged as an outcome of the inductive content analysis of the data (nine from INT2 and three from INT3). Themes TU1-TU11 were developed from interviews with clinicians, while theme 12 emerged from the data gathered from women with GDM. The findings are presented below and in Table 3, where each theme is illustrated with relevant excerpts from the interview data.

1. Theme TU1 (sharing patient data with the clinical team): the system displays, together, blood glucose readings, insulin dosage, food diary and exercise data, which saves time looking for pieces of information from various sources.
2. Theme TU2 (combining food intake data and blood sugar data): the system receives input about the food intake and glucose readings on daily basis, stores the data in a single database and generates a combined report (not possible given the current hospital systems).
3. Theme TU3 (pattern identification in combined data): women with GDM maintain frequent contact with midwives but have limited access to a dietician (only if their condition deteriorates). The system presents an integrated view of patient data that allows for the identification of odd patterns and provides support for subsequent consultations.
4. Theme TU4 (downloading accurate data): for a number of reasons, a woman with GDM may try to conceal a high BG reading by replacing it with a lower value. Automatic downloading removes the opportunity to manipulate BG readings.
5. Theme TU5 (patient self-awareness): self-management is facilitated by being aware of the current measurements of the factors related to their condition, such as blood sugar level, diet and weight.
6. Theme TU6 (help with food recall): with mobile apps, women with GDM will be able to complete food diary entries immediately rather than retrospectively. This will improve the accuracy of the shared data.
7. Theme TU7 (essential nutrients required): certain nutrients such as iron, calcium and protein are important for pregnancy and should be part of the data. However, women may not be experts in identifying the nutrients in their food diary. Hence, they should record everything they eat to allow the dietician to analyse their diet and complete a nutrition assessment.
8. Theme TU8 (time saving): it saves time when a physician, obstetrician or dietician can see the patient without the need for the physical file held by the midwife. Additionally, most of the data entry is completed by women in their own time, with no need for clinicians to feed data into the hospital systems retrospectively (and potentially, not accurately (once the ecosystem prototype is integrated with the hospital system)).
9. Theme TU9 (remotely manage patients): once educated about managing the device and apps and able to self-manage, women with GDM will need to visit the clinic less frequently. Clinicians can access data about women’s conditions and recommend a diet or insulin dosage change.
10. Theme TU10 (educating women about carbohydrates and insulin dosage): women with GDM have to learn how to manage their condition in a short timeframe. It helps them not to need to count carbohydrates and record food in grams but rather to keep track of food intake measured in cups or other convenient units and still be able to adjust insulin dosage.
11. Theme TU11 (food diary): it may be impractical to go through each day’s food diary and extract information about nutrient values from food databases. Women could self-manage this in their own time if the prototype had an interface for the women (especially as food intake entries are only checked if their sugar levels are outside the norm).
12. Theme TU12 (willing to accept): the proposed system will be useful, as clinicians can see all information together in one place. The system will be especially useful for women who need clinical advice on changing their diet or insulin dosage. Specific benefits include logging food intake anytime and anywhere (helpful for food recall and allows one to feed inaccurate data) and enabling dieticians to make diet changes on a daily basis.

The themes emerging from the INT2 and INT3 interviews with clinicians (themes TU1-TU9) reinforced the findings of the analysis of the INT1 data and indicated that clinicians found the system useful and beneficial to their everyday practice. In particular, clinicians found the system useful in addressing current practice deficiencies (themes TU1, TU2 and TU3), providing reliable data (themes TU4, TU6, and TU7), empowering women with GDM (themes TU5 and TU10), and empowering clinicians (themes TU8, TU9 and TU11). The views of women with GDM as represented in Theme TU12 are aligned with clinician views, more specifically as expressed in themes TU1, TU2 and TU 6, and indicate that women with GDM are willing to accept and use the system.

### 3.2. User Evaluation of System Usability/Ease of Use 

User views about the usability/ease of use of the ecosystem prototype were gathered at the TA sessions TAP1 and TAP2. During TAP1, the five participating clinicians were asked to complete two structured tasks around viewing the combined wellness report for a specific patient and a selected data range and importing wellness data. During TAP2, participants (10 clinicians, including the ones who took part in TAP1, and five women with GDM) were asked to first complete the same two tasks as at TAP1 and proceed with the new structured tasks. The new tasks focused on improving the semantic meaning of wellness data by adding relevant clinical terms following appropriate standards. This included identifying fasting (pre-prandial) and non-fasting (post-prandial) readings and assigning an appropriate LOINC code, assigning SNOMED CT codes to food items and assigning nutrient value. Fasting and post-meal blood glucose readings were already flagged by the glucose meter; this was programmatically changed in the prototype to include the LOINC code for pre- or post-prandial blood glucose readings. SNOMED CT terminologies are used to denote food mainly for allergies. In the ecosystem, SNOMED CT codes were used to identify food if required by clinicians (especially dietitians), for example, if some food entries needed to be clinically represented for transmitting to clinical systems. As in TAP1, all tasks included finding a specific patient and updating patient data for a specified period. 

The TAP1 and TAP2 data were examined and coded inductively. Participant views about the usability/ease of use of the system were captured in the seven emerging themes. The data were interpreted and coded inductively. The first five themes (TE1-TE5) were based on the analysis of the data gathered from clinicians. Themes TE1-TE4 capture clinicians’ views expressed during the TAP1 session, with themes TE2 and TE4 developed further from TAP2 data, which also rendered a new theme (TE5). Theme TE6 was derived from data gathered from women with GDM (session TAP2). The themes are described below, and in Table 4, which provides data excerpts illustrating each theme.

1. Theme TE1 (navigation): the participants associated the prototype with systems used in the clinic and commented on the navigation with reference to their prior experience. For example, when completing a task and moving to the next one, most participants attempted to ’find’ the patient again even though this was not needed. Similarly, participants could not always follow the correct order of actions (represented by buttons) needed to complete the task.
2. Theme TE2 (system output): participants suggested to redesign the screen layout (where possible) to facilitate quick reading. For example, the system output (the combined report) was planned to display meal times in a 24-h column format since women with GDM need to take their glucose readings with respect to meal times (that are not fixed). Five meal timing sections were designed, to accommodate breakfast from 01:00 to 10:00 morning tea from 10:00 to 12:00, lunch from 12:00 to 15:00, afternoon tea from 15:00 to 17:00 and dinner from 17:00 to 24:00. Blood glucose readings were to be displayed in the hourly columns, close to the food diary data. A similar approach was to be followed for displaying exercise data. However, compared to the format in which blood glucose and insulin readings were displayed, food data entries were rather longer; the hourly columns in the report were narrow and the food descriptions normally broke into multiple lines (e.g., a chicken sandwich is described as two brown bread slices, 25 g of chicken, 1 tablespoon of mayonnaise sauce, 1 tablespoon of mustard sauce, rocket leaves, 2 slices of tomato and 2 tablespoons of mashed avocado). Participants found that the detailed record displaying each and every ingredient of the food item was too hard to follow. An alternative would be to ‘hide’ the ingredient description and display it when a mouse hovered over it (but losing the ability to compare the ingredients of different food items). As a result of these issues above, viewing the system output (the combined report containing the blood glucose, insulin dosage, food diary and exercise data) was rather hard as it did not fit in one screen. Reading the report required significant scrolling. Another issue was the 24-h format (e.g., ‘from 15:00 to 17:00’ meaning ‘from 3:00 p.m. to 5:00 p.m.’). To resolve the readability problems, the time slots were re-labeled and the columns were made re-sizeable; a pdf version of the report was added. Users found that the pdf file was easy to scale on the screen and the user could easily view all data for the day across one screen. Another impediment to the screen readability was the insufficient contrast, but this issue was not addressed by the developer.
3. Theme TE3 (feedback): in general, clinicians found the system easy to use as its functions were aligned with their everyday work practice and environment. However, the labels on some of the navigation buttons confused them: an interesting example was the ‘Back’ button, which needed to be pressed after completing a task. Participants perceived moving to the next task as going forwards, not backwards. They also felt the need to receive system feedback on completing a task (acknowledgment or confirmation). Users found the error messages generated by the system easy to understand and follow, helping one to continue with the task at hand; adding more guidance on the screen would have been helpful too.
4. Theme TE4 (terminology): clinicians were at ease with the medical terminology used in the system interface but found some of the ‘IT’ terms less than intuitive. For example, the options to ‘Export data’ and ‘Import data” were hard to interpret correctly, and this slowed down the completion of the related tasks. Once participants got more familiar with the system functions, they accepted some of the terms, for example, ‘Import data’ was retained as it was correctly understood as transferring data from external devices, to the system. The ‘Export data’ option data was considered better named as ‘Report’ as it referred to generating the combined report that consolidated data from various sources. Another major impediment to completing the tasks was that clinicians were not used to annotate patient generated using medical terminology such as LOINC codes.
5. Theme TE5 (comments): comments were useful for clinicians to make notes alongside the data readings. Some of the comments would reflect the activity, food intake or insulin dosage.
6. Theme TE6 (existing experience): women with GDM who reviewed Prototype 2 did not have much feedback to offer about the interface and design as the system was built primarily for clinicians. However, most participants were able to complete the first three tasks without difficulty as they were familiar with computers and smart phones.

With the ‘live’ prototype, participants found it easy to discuss how the system worked and comment on details they perceived as important. The first four themes (TE1–TE45) captured usability issues related to the system interface (system output, onscreen navigation, system messages and guidance, and clinical and IT terminology). Users positively accepted the improvements in the interface made in Prototype 2, especially on the improved formatting of the system output; clinicians already involved with the self-management of Type 1 diabetes patients gave particularly useful feedback. Theme TE5 shows how enhancing the interface by adding an easy-to-use space for entering comments improves as well the perceived usability of the system. Theme TE6 indicates that women with GDM might be comfortable using a patient interface if one were to be developed. 

## 4. Discussion

The iterative nature of the prototype development with an UCD approach helped to achieve a deeper understanding of the GDM consultation context and the required functionalities of the proposed clinical ecosystem. The prototype design and development process included multiple user evaluation rounds that focus on the ecosystem’s perceived usefulness and usability/ease of use, following the TAM assumptions about the positive impact of perceived system usefulness and ease of use on user acceptance. The evaluation activities sought to examine the acceptance of a clinical system that integrated the wellness data required for the self-management of GDM and focused on the robustness of the software prototype and the integration of heterogeneous data from various mobile apps. 

The clinical research participants (obstetricians, a physician, midwives and dieticians) were enthusiastic about the project and were happy to support the research. The prototype demonstrations and the TA protocol sessions gave ample opportunities for hands-on engagement with the system and provided meaningful feedback. The semi-structured interviews were also very informative as participants proactively discussed the systems concepts and functional capabilities. The two data gathering modes complemented each other and provided a comprehensive picture of the views of the participating clinicians about the system’s usefulness and usability. 

Enabling women with GDM to collaborate with the clinical team in the management of their GDM condition was a strategic objective for the clinic hosting the research, leading to improved communication between women with GDM and midwives, and improved clinical outcomes. Hence, women with GDM were included in the later stages of the prototype development. Their input was equally important in understanding the combined report generated by the prototype as this was intended to be used at consultation time with midwives.

While clinicians participating in the first round of interviews found the proposed clinical ecosystem useful and saw value in developing a software prototype, the paper mockup prototype that was used failed to engage participants. As a result, very little feedback was obtained about system navigation and functional requirements. Hence, better approaches were thought of in the subsequent collaboration with clinicians to build the system. Developing the software prototype and having clinicians participate in tasks related to the prototype clinical ecosystem functionalities and verbalise their impressions and perceptions (using the TA protocol) brought out the pertinent usability and ease-of-use issues. 

The twelve usefulness themes derived through the inductive content analysis of the semi-structured interviews and the six usability/ease-of-use themes identified through the two TA sessions indicated that the research participants (clinicians) were highly motivated and interested in developing a clinical system suitable to their everyday practical needs. Similar usability themes around system interface such as navigation, screen layout, feedback and terminology have been identified in other usability studies [35]. However, this prototype was developed for a specific context and relied on user feedback about the menu options, the wellness data display format, the error messages and the use of clinical terminologies. For example, LOINC and SNOMED CT coding were used to improve the semantic meaning of the data (the purpose of using LOINC was to identify pre- or post-prandial blood glucose readings, while SNOMED CT was used to identify dietary items). However, not all clinicians were sufficiently familiar with them, and it may have been more appropriate to make their use optional. On the other hand, dietitians were able to advise on how to incorporate and represent food diary data for the purposes of self-management (as some of the apps did not have a built-in database with food item names, descriptions and calories and only allowed for user provided food intake descriptions). To address the issue, the clinical ecosystem prototype included an option to import the missing nutrition values from external sources such as the New Zealand Food Composition Database (https://www.foodcomposition.co.nz/, accessed on 10 December 2021).

While the main functional requirement was to integrate wellness data from the mobile apps and the glucose meter, throughout the prototype evaluation activities it became clear that the order of the columns in which the data were displayed was also very important. Clinicians who were using a software system (Diasend) for the management of Type 1 diabetes were accustomed to reading the data values in a particular order.

Clinicians expected that the system output would help identify emerging ‘cause and effect’ patterns related to the patient’s GDM condition, such as relationships between food intake and glucose reading. They provided some specific requirements regarding the visual display of the report data on the screen to enable an easy reading and value comparison at consultation time, such as the food column being displayed before the post-prandial glucose reading. Thus, the original functional requirement had to be extended to improve the usability of the implementation of this functional requirement and to support efficient operational use. For example, the way data were presented (the column order) helped the consulting clinician to quickly grasp the meaning of the pattern formed by the values displayed. Ultimately, the data display and the user interface needed to provide effective support for the clinical analysis (ecosystem usefulness), while reading and working with the data needed to be easy and efficient, without unnecessary delays (ecosystem usability/ease of use). 

While clinicians were the primary system stakeholders and the system interface was designed to meet their requirements, women with GDM were able to view their own data on the system screen at consultation time. The data analysis indicated that that the system interface design had to make provisions for patients to enable them to understand the system output and discuss it with the consulting health professional. 

The research question of the study was formulated as “How to ensure that the system design process of a clinical ecosystem that integrates patient-generated wellness data facilitates user acceptance?” The outcomes of the research indicate that the target users perceived as useful the ecosystem described and that the UCD design process in which target users were involved in the iterative system design and development was instrumental in meeting user expectations about the ecosystem’s usability/ease of use. Both clinicians and women with GDM accepted the concept of receiving wellness data from patients’ mobile apps as a useful means of correctly recalling and entering wellness data, and improving the communication between clinicians and women with GDM. The feedback from the research participants in clinical roles who took part in identifying the main requirements and improving the system output indicated their acceptance of the patient report, which combined wellness data from various sources such as a glucose meter and mobile apps keeping track of food intake and exercise. The research contributed by developing a working prototype of a clinical system able to provide to consulting clinicians all the data they need in one consolidated report, without having to peruse different sources.

Further development of this project may include a pilot implementation of the clinical ecosystem (Prototype 2) and refining the design with a long-term goal of making the system part of the family of integrated hospital systems. The provision to code patient data stored in the integrated database using health informatics standards for non-clinical data (LOINC and SNOMED CT) is already in place; exchanging health record using protocols based on fast health interoperable resources (FHIR) will facilitate the proposed ecosystem’s interoperability with other relevant clinical systems. 

It was envisaged that at the implementation stage, a strong data security and privacy protection layer would be developed in alignment with the implementation context such as role-based access control for clinical staff access and applying an anonymisation protocol to data extracted for the purposes of aggregation/reporting or research. In line with current practice, it is not anticipated that women with GDM would be given access to data stored in the database of the clinical ecosystem; however, individual patients can view their data in the wellness app they use. 

The clinical ecosystem developed as a prototype works with a limited number of mobile apps. Furthermore, the participant sample was drawn from the staff and patients of one particular clinic; in particular, age and experience were not considered as a selection criterion but may be factors influencing ease-of-use perceptions. To address these limitations, further research will need to consider the views and opinions of women with GDM from a wider range of socio-economic and cultural background. Recruiting clinical staff from a range of relevant health provision units, and from different professional backgrounds, would be particularly helpful in refining the interface design. Collating data from multiple device types and a number of different mobile apps and various devices will continue to represent a challenge. Further ecosystem development may include creating and implementing a framework for the selection of appropriate mobile apps that takes into account both technological and clinical requirements. 

## 5. Conclusions

In this study, a UCD approach was adopted and applied to the development of an mHealth system prototype. The system was developed iteratively, with user feedback gathered at every iteration. The multimodal approach to gathering user feedback involved TA protocol sessions, prototype demonstrations and semi-structured interviews. The qualitative content analysis sought to developed themes around the TAM variables PU and PEOU. The approach towards the design and development of the clinical ecosystem was successful in producing a prototype that met stakeholder needs and requirements and contributed an innovative mHealth system. 

## Figures and Tables

**Table 1:** Study sample characteristics and participant involvement.

Role	Profile	Involvement
Dietician	Nutrition assessment of patients with gestational diabetes	Semi-structured interviews INT1, INT2 and INT3. TA sessions TAP1 and TAP2
Obstetrician	Provides care during pregnancy and birth and deals with complications caused by GDM	Semi-structured interviews INT1, INT2 and INT3. TA sessions TAP1 and TAP2
Midwife	Provides primary care related to GDM during pregnancy, monitors weekly/fortnightly progress.	Semi-structured interviews INT1, INT2 and INT3. TA sessions TAP1 and TAP2
Woman with GDP	Pregnant woman diagnosed with GDM, with no prior diabetes condition	Semi-structured interview INT3. TA session TAP2

**Table 2:** System evaluation stages.

	Ex-/Post-Ante	Evaluation Material	Evaluation Method	Participants	Main Focus
1	Ex-ante	Mock-up prototype on (paper)	Structured interview INT1	Clinicians (5)	Identifying key user needs/requirements for a useful clinical system
2	Ex-ante	Prototype 1	Semi-structured interview INT2	Clinicians (5)	Review of system functionality. Initial feedback on system interface and navigation
3	Ex-post	Prototype 1	TA tasks: session TAP1	Clinicians (5)	Examining system usability and ease of use
4	Ex-post	Prototype 2	Semi-structured interview INT3	Clinicians (10), women with GDM (5)	Further review of system functionality; gauging user acceptance
5	Ex-post	Prototype 2	TA tasks: session TAP2	Clinicians (10), women with GDM (5)	Further examination of system usability and ease of use; gauging user acceptance

**Table 3:** Data excerpts supporting system usefulness themes.

Theme	Interview Excerpts
TU1	“The prototype helps share data within the team and I do not have to ask the midwife for the patient file”
TU2	“Yes, it includes pretty much everything that you want to know about: BG ^1^, FD ^1^, exercise”; “You need an app which gets the calories, carbohydrate breakdown, protein and fat”; “Wellness data is representative. Easy to compare FD, exercise, insulin dosage in comparison with BG”; “Reflects physical activity and FD together with treatment and dosage and sugar level”
TU3	“I can see people’s pattern of their eating and timings as well”; “You notice the frequency and when people are doing something different which is part of the learning and teaching we give a lot of information”; “…Useful, I see the patients once, and could just run an eye over the report thereafter even if I don’t have an appointment”
TU4	“BG readings are fudged to please the midwife”; “The issue with patients using apps is about accuracy and telling the truth”; Patients do not like having their health records saved specially when they have high readings”
TU5	“Self-awareness help women keep their sugar and their weight under control”
TU6	“App could help with recall…”; “Patients should have knowledge of what they are eating and record it correctly in the app”
TU7	“Calories will not tell you what you have eaten… I want to know everything they are eating”; “What’s the carbohydrate amount is important, also accessing them for nutrient adequacy”
TU8	“When a woman comes to my room, I don’t have to find a midwife what she said to her and we don’t waste time”
TU9	“We can all log into our computers and access the information about the patient whom we want to see”
TU10	“I know my patients would find it helpful to know how many carbohydrates”; “It may help quantify how much insulin to take or adjust to… it is sufficient for them to say 2 slices brown bread, ½ cup rice”
TU11	“It is nice that it incorporates with food database… But the amount of time we get to be with the women there is probably not much time”; “They only need to write down only if they have been eating only for that time what they have had high blood sugars”
TU12	“I would absolutely use it and comfortable to share the data with a clinician if suitable apps are available”; “…data is available for clinicians at their fingertips. It can be entered into the system directly, taking out the need for unnecessary paperwork which can be time consuming. Also once data is available it can be analysed in various ways for the betterment of patients suffering from gestational diabetes”; …I think probably 70% of people don’t mind, they don’t mind, you know, to be honest”

^1^ BG: blood glucose; FD: food diary.

**Table 4:** Data excerpts supporting system usability/ease-of-use themes.

Theme	Interview Excerpts
TE1	“Sometimes for our bookings for … ultrasound say, so we have got one screen then next another screen, we click next and it is self-explanatory”; ”It looks like it finishes there (on screen seeing part of the data). So it is not very good”
TE2	“Text in narrow column breaking into multiple lines”; “Challenging if all details are entered, difficult to fit in narrow columns”; “(Viewing the pdf) This is much nicer to look at. You can see what exactly the readings were, when the readings were taken and what has been eaten with different meals on different days. It is much easier to see in pdf than the first report that was generated (on screen)”; “Time in hours as heading across is ideal to read”; “Easy to compare FD, exercise, insulin dosage in comparison with BG”; “Hard to read the update on the blue background”; “I think colour scheme and it is not just black and white. Maybe it is easier to read”; “Glucose readings across the day with carbohydrate intake is preferred”
TE3	“Easy. Should not be difficult as it is work related”; “I might have little problem in the beginning about navigation. How to move forward when the button on screen says ‘Back’”; “Once you have learned the navigation and menu it won’t be a problem”
TE4	“I have clicked one reading and not sure what LOINC readings are?”; “It is pretty user friendly”; “I go edit and use drop down menu to select one code fasting and non-fasting?”
TE5	“I might write a comment saying if I know she forgets her insulin, she did more exercise than usual, or she had a big slice of cake (baby shower), maybe that kind of thing I might write.”
TE6	“Most women don’t mind doing email. Most women are on email so emailing blood/sugars… I think probably 70% of people don’t mind, they don’t mind, you know, to be honest…”; “…used Fitness pal, step counter”; “And if you offer them an app I am sure the young ones especially would be [interested]…”

## Data Availability

Data supporting reported results are available on request.
